# X-ray diffraction tomography with limited projection information

**DOI:** 10.1038/s41598-017-19089-w

**Published:** 2018-01-11

**Authors:** Zheyuan Zhu, Alexander Katsevich, Anuj J. Kapadia, Joel A. Greenberg, Shuo Pang

**Affiliations:** 1 CREOL, The College of Optics and Photonics, University of Central Florida, Orlando, 32816 USA; 2 Department of Mathematics, University of Central Florida, Orlando, 32816 USA; 3 Department of Radiology, Duke University, Durham, 27705 USA; 4 Department of Electrical and Computer Engineering, Duke University, Durham, 27708 USA

## Abstract

X-ray diffraction tomography (XDT) records the spatially-resolved X-ray diffraction profile of an extended object. Compared to conventional transmission-based tomography, XDT displays high intrinsic contrast among materials of similar electron density and improves the accuracy in material identification thanks to the molecular structural information carried by diffracted photons. However, due to the weak diffraction signal, a tomographic scan covering the entire object typically requires a synchrotron facility to make the acquisition time more manageable. Imaging applications in medical and industrial settings usually do not require the examination of the entire object. Therefore, a diffraction tomography modality covering only the region of interest (ROI) and subsequent image reconstruction techniques with truncated projections are highly desirable. Here we propose a table-top diffraction tomography system that can resolve the spatially-variant diffraction form factor from internal regions within extended samples. We demonstrate that the interior reconstruction maintains the material contrast while reducing the imaging time by 6 folds. The presented method could accelerate the acquisition of XDT and be applied in portable imaging applications with a reduced radiation dose.

## Introduction

Due to its high penetration power, X-ray-based imaging is ubiquitous across research and applications areas including material science, geology, medical imaging, pharmaceutical science, non-destructive testing, and security screening^1–5^. Small angle X-ray scattering (SAXS) is a major probe of the structural information at the nanoscale and is widely used in solving crystalline structures, phase identification, and mass fraction estimation^6,7^. For heterogeneous samples, scanning X-ray diffraction imaging scans a pencil X-ray beam through the object to record a scattering pattern at each point, which combines the ability of SAXS as structural probe with spatial resolution, but it does not provide depth information^8–10^.

Since the diffracted X-rays deviate from the incident beam, depth of the scatter along the beam can be resolved with specifically-designed encoding patterns on either the illumination side^11–13^ or detector side^14,15^. However, due to the small scattering angle, the achievable depth resolution of these methods is on the order of 10 mm. To overcome this limitation, X-ray diffraction imaging based on computed tomography (CT) approach has been proposed^16^. Synchrotron-based X-ray diffraction tomography (XDT) systems have been used for imaging fibrous collagens and identifying crystalline compounds^1,17,18^. However, limited access to synchrotron facilities excludes many real-life imaging applications. Table-top fan beam diffraction systems, termed coherent scattering computed tomography (CSCT), were proposed for breast cancer screening and security imaging applications^19,20^. These setups, relying on the detector-side collimation to localize the diffracted X-ray beam, achieve a resolution of several millimeters. However, due to the low collection efficiency of the collimators, full CSCT scan requires tens of hours of imaging time and results in a high radiation dose to the sample. For many X-ray imaging applications, especially in clinical and security fields, X-ray diffraction is ideal for secondary scan^21,22^, and scanning the entire object is not necessary; a region-of-interest (ROI) reconstruction displaying high material contrast is desirable.

Here we propose a table-top X-ray diffraction tomography method that reconstructs the selected ROI, which reduces the image acquisition time while preserving the material specificity from diffraction profile. One order of magnitude of dose reduction compared to full sample scan was demonstrated.

## Results

### Interior XDT reconstruction of a phantom

**Figure 1:**
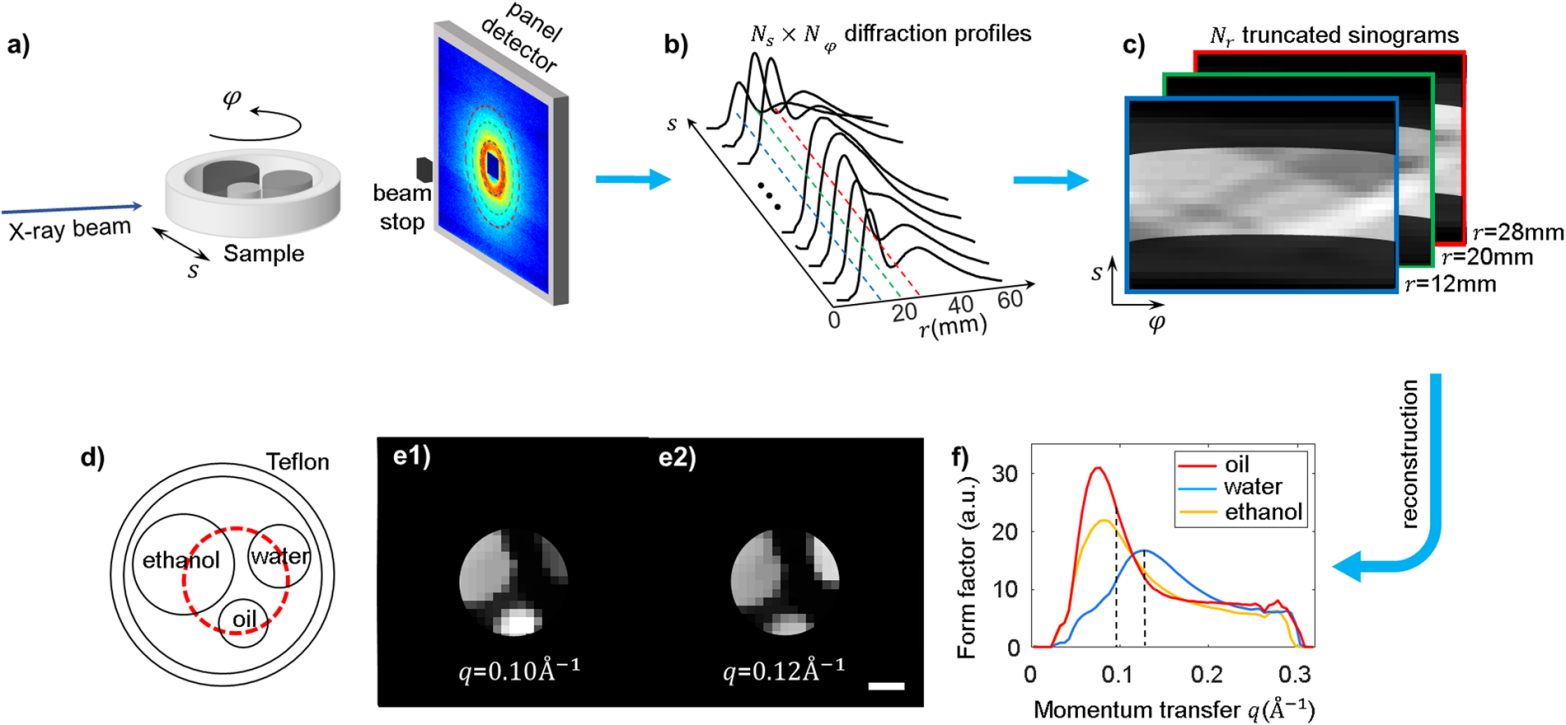
Tomography system setup and reconstruction. (**a**) Illustration of the X-ray diffraction tomography (XDT) system. The sample is scanned across the pencil beam and rotated. A 2D diffraction pattern is captured at each sample position. (**b**) By averaging the 2D diffraction pattern in the azimuthal direction, the intensity profile along the radial direction, $r$, is extracted. (**c**) Each radial position along the diffraction profile forms a projection sinogram. For interior scan, the projection data does not contain translation outside the ROI. (**d**) Illustration of the sample composition. The red, dashed circle marks the ROI region. (**e**) Interior XDT reconstruction of the phantom from truncated projections. Different contrasts can be observed among water, ethanol and Smucker’s® soybean oil at momentum transfer $q$ = 0.10 ${\mathring{\rm A} }^{-1}$(e1), and 0.12 ${\mathring{\rm A} }^{{-}1}$ (e2). (**f**) Reconstructed form factor profile of water, ethanol and oil within ROI. The scale bar represents 5 mm.

A phantom consisting of ethanol, water, Smucker’s® soybean oil, and Teflon was analyzed by the table-top XDT system, as shown in Fig. 1(a). The sample was illuminated with a pencil beam collimated by a pair of 2 mm pinholes. For each rotation and translation pair, $(\phi ,{s})$, a 2D diffraction profile of the sample was measured. Each 2D profile was segmented into a series of rings along radial position $r$ and transformed to linear diffraction patterns ${g}_{\phi }(s,r)$, as shown in Fig. 1(b). Each radial position $r$ along the diffraction profiles were extracted to form one projection sinogram, as shown in Fig. 1(c). The stack of 2D diffraction profile is thus rearranged to a 3D dataset whose dimensions are position $s$, rotation angle $\phi$ and radial location $r$. If the whole sample is scanned along the $s$ direction, we get the whole set of sinograms. The region of interest was selected by limiting the translation range during the acquisition process. The diameter of the phantom is 32 mm, and the ROI was selected at the center with a diameter of 14 mm (Fig. 1(d)). The sample was rotated with a step size of 5° covering the total 180° for both global and interior scans.

For the global scan, a reconstruction method based on filtered backprojection has been demonstrated to recover the spatial distribution at each momentum transfer value separately^1^. However, this is only valid when the detector to sample distance is sufficiently large. For table-top systems, we establish that 2D XDT scan can be treated as 3D circular parallel fan beam computed tomography (CT), (**SI** Sec. 1), and classic reconstruction algorithm for cone beam CT can be applied (**SI** Sec. 2). For truncated projections, unique and stable reconstruction is a topic that has attracted plenty of research efforts^23–25^. Here we exploit the piecewise smoothness of our sample, and use total variation regularization to achieve stable reconstruction^25,26^. Different contrasts are displayed in the reconstructed images at momentum transfer $q$ = 0.10 ${\mathring{\rm A} }^{-1}$, and 0.12 ${\mathring{\rm A} }^{-1}$ (Fig. 1(e)), due to the distinctive molecular signatures of form factor profile (Fig. 1(f)). The normalized mean square root difference inside the ROI between global and interior reconstruction is 0.9%.

### Material Classification

XDT provides superior contrast when compared to conventional absorption CT (**SI** Sec. 3). We further demonstrate the interior XDT reconstruction maintains the material contrast. Specifically, the reconstruction of the spatially-resolved form factor within the ROI can successfully identify the material distribution based on the point X-ray diffraction profiles.

**Figure 2:**
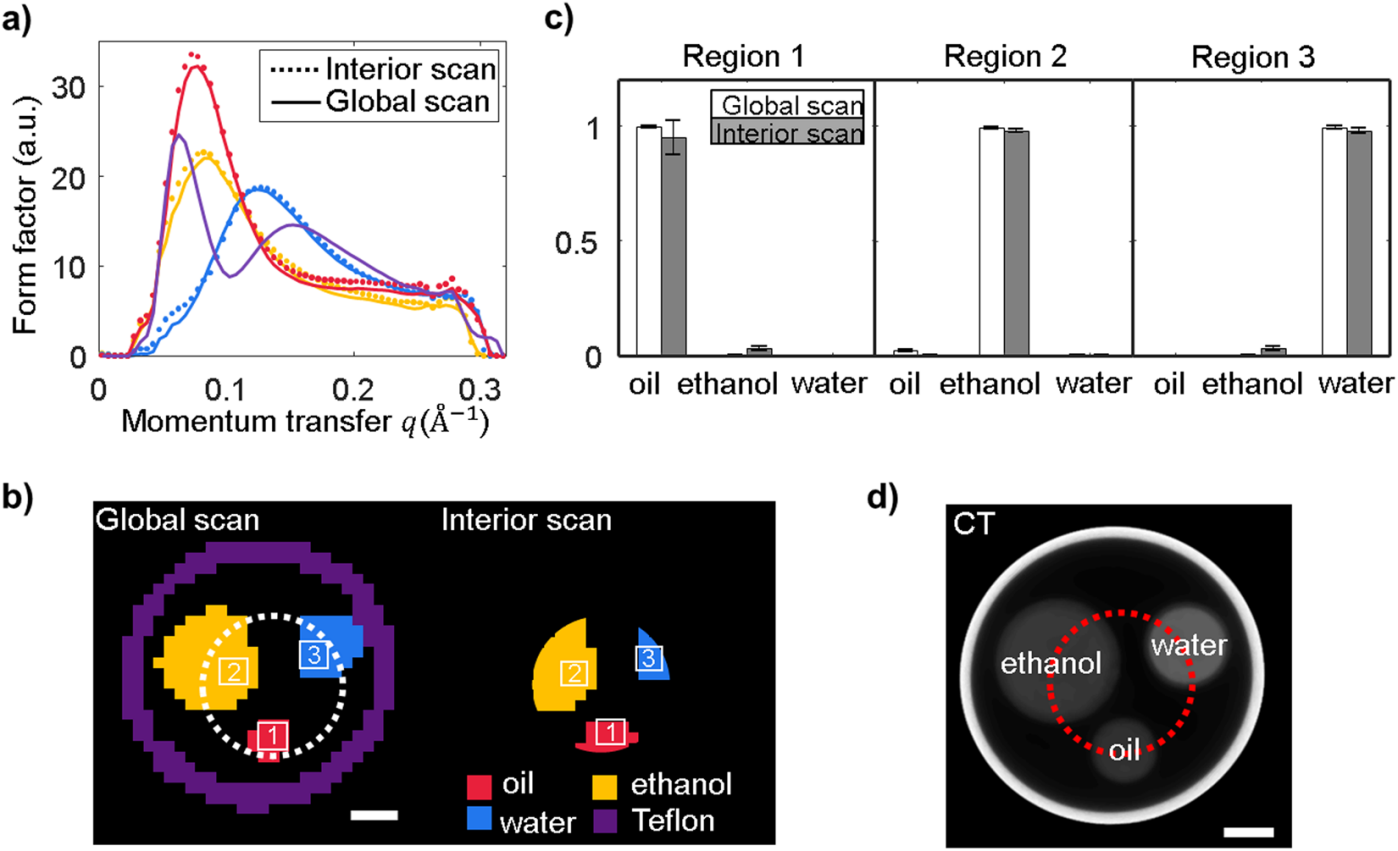
Comparison between the reconstruction from full-FOV and interior XDT scan. (**a**) Reconstructed form factor from global and interior XDT scan compared with reference form factor of each material. (**b**) Material map of the global and interior scan. (**c**) Classifiers in boxed regions marked by the numbers in (**b**). The bin and error bar indicate the average and standard deviation of the probability within each region. (**d**) Conventional CT reconstruction. The scale bars represent 5 mm.

The reconstructed form factor profiles $f(q)$ from the truncated projections are compared to the single point X-ray diffraction profiles (Fig. 2(a)). The material classification of the interior reconstruction was performed using support vector machines (SVMs)^27^, which construct optimized hyperplanes to discriminate high-dimensional form factors into five material classes. These trained SVMs were first tested to classify the global XDT reconstruction (**SI** Sec. 3.1). The trained SVMs were then applied to classify the form factors from the interior XDT reconstruction. Among all five SVMs, the class that yields the highest score was identified. The material classification map for both full-FOV and interior XDT reconstruction was shown in Fig. 2(b), which shows little discrepancy between the interior and global reconstruction. We picked three regions, each composed of 3 by 3 pixels, containing one of the three materials in both interior and global reconstruction. For the convenience of comparison, the SVM score was transformed to a probabilistic output. The probability of each pixel belonging to a specific class was compared in Fig. 2(c). The probability of the pixels in Region 1 being soybean oil is 0.948 and 0.995 for the interior scan and global scan, respectively; the probability of Region 2 being ethanol is 0.981 and 0.991 for interior and global scan, respectively; the probability of Region 3 being water is 0.978 and 0.991 for interior and global scan, respectively. The results showed excellent agreement with the ground truth. We speculate that the greater difference in the form factors in Region 1 is due to its proximity to the ROI boundary. This effect has been observed in interior tomography due to the increasing error in reconstruction close to the boundary^28^.

By measuring the complete diffraction profile rather than the transmittance, the XDT reconstruction particularly improves the contrast between soybean oil and methanol, which would be indistinguishable in a traditional CT image (Fig. 2(d), **SI** Sec. 3). Compared with the global XDT scan, the interior scan retained the material contrasts while reducing the total acquisition time by 2.5 times.

### Interior XDT reconstruction of biological tissue

**Figure 3:**
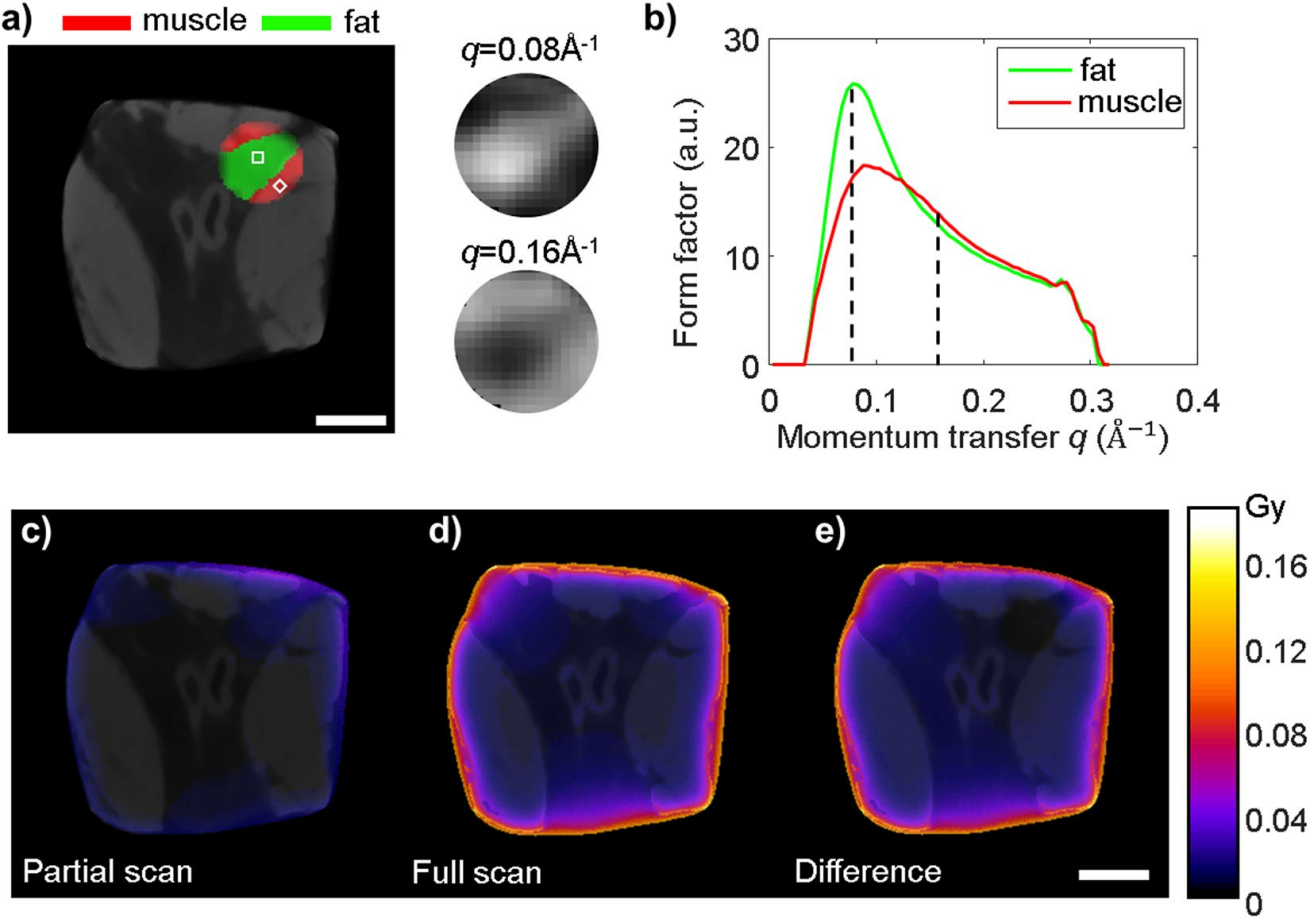
(**a**) Interior XDT reconstruction with fat and muscle region superimposed with a CT image of the sample. The inset shows the XDT contrast between fat and muscle at $q$ = 0.08 ${{\rm{\mathring{\rm A} }}}^{-1}$ and 0.16 ${{\rm{\mathring{\rm A} }}}^{-1}$. (**b**) Reconstructed diffraction form factor of fat and muscle inside the ham sample. (**c**) Simulated absorbed dose of interior XDT scan. (**d**) Simulated absorbed dose of global XDT scan. (**e**) The difference in absorbed dose distribution between the global and interior scan. The total dose administrated to the sample is reduced by 83%. The circle marks the interior region. The scale bar represents 5 mm.

The advantages of interior XDT method are its accelerated imaging speed and the reduced radiation dose. We have demonstrated the interior imaging of biological tissue by reconstructing an ROI with diameter of 5 mm inside a 23 mm × 23 mm pork tissue (Fig. 3(a)). Two types of tissues corresponding to fat and muscle in the ROI are distinguishable from the truncated measurement. The X-ray diffraction profile (Fig. 3(b)) of muscle resembles that of water, while fat gives a strong diffraction peak at lower momentum transfer due to the long chains of lipid mono-layer, which is consistent with the previously reported results^29,30^. The resulting material map of the ROI is shown in Fig. 3(a), with fat rendered in green and muscle rendered in red. A CT image of the pork sample is shown in the background for reference. The interior XDT scan acquisition time was 2.3 hours, while the global scan of the whole sample would take 13.4 hours.

We investigated the difference of the absorbed radiation dose between global and interior XDT scans through Monte Carlo simulation (Fig. 3(c–e)). The radiation doses inside ROI are comparable in both cases, since in both scenarios, the interior region absorbs radiation from all rotation angles. However, in exterior region, interior XDT scan significantly reduced the administrated dose, showing an average dose reduction of 83% when compared with global scan.

## Discussion

The aim of the two described examples is to show the capabilities of interior XDT as a non-invasive high contrast tomography technique using a table-top X-ray source with accelerated imaging speed. Compared with fan beam CSCT, which requires detector collimation, the demonstrated system requires collimation on the source side. The throughput of both systems is similar for global scan. The interior scan in fan beam CSCT with detector side collimation could be implemented by reducing the extent of the fan beam illumination. However, though the radiation dose would be reduced, an interior scan would not shorten the acquisition time. The pencil beam XDT system has demonstrated a 6-fold reduction in acquisition time for the reconstruction of an interior area ~10% of the whole object.

Another asset of our proposed system is the possibility to combine simultaneous X-ray diffraction with fluorescence and absorption^31^. The demonstration illustrates the capability of XDT and allows us to extract information that is more conclusive in terms of the classification of chemical composition. Both diffraction and fluorescence signals are much weaker than transmission. Similar to CT development, further parallelization in acquisition process would efficiently improve the acquisition time. It is not unreasonable to assume that such approaches will supersede the more traditional ‘single point’ *in situ* methods for studying many industrial and medical processes.

## Methods

### XDT setup

X-ray diffraction tomography system employs a filtered copper-anode X-ray tube (XRT60, Proto Manufacturing) as the quasi-monochromatic source. The emission peak is centered at 8.0 keV. The tube operates at 35 kV and 40 mA. The focal spot size of the tube is 0.4 mm. The emitted X-ray was collimated by a pair of pinholes, each 2 mm in diameter and separated 100 mm apart, into a pencil beam. The distance from the source to the first pinhole was 260 mm, and that between the second pinhole and the sample was 60 mm. This collimation geometry created a 2.4 mm-diameter focal spot on the rotation plane. The sample was mounted on a rotational stage (RV1200P, Newport) and a linear stage (UTM150CC, Newport) for tomographic projection. The step size of the linear stage was 1 mm, matching the Nyquist sampling. The projection over 180° rotation was uniformly covered by 46 rotation angles with a step size of 4°. Diffracted X-rays from the sample were captured by a panel detector (1215CF-MP, Rayence), which was placed 120 mm away from the center of rotation. The detector operated on 4× binning mode, a total of 588 × 736 pixels with effective pixel size of 0.2 mm. The central 10 × 10 mm region on the detector was blocked by a lead beam stop. The acquisition time for each projection was 45 seconds. Background images were captured after each rotation step. For comparison with conventional computed tomography, a CT scan covering projection angles from 0 to 180° at a step of 1° was also performed on the same sample using parallel-beam geometry.

### Reconstruction methods

After preprocessing, the set of sinograms is a 3D function $g(r,s,\phi )$, where $r$ is the distance between detector pixel and pencil beam, $s$ is the beam offset, and $\phi$ is the projection angle. Since the diffracted photons originate from both interior and exterior region, the full size of the object ${\bf{f}}$, rather than the interior region, was discretized. For global XDT, a modified Feldkamp-David-Kress (FDK) algorithm for parallel fan beam geometry was used in reconstruction^32^. For interior reconstruction, the forward matrix ${\bf{H}}$ establishes the linear mapping between the object and the vectored truncated measurement ${\bf{g}}$. The object **f** is reconstructed by a maximum-likelihood estimator^33^ with TV regularization^34^. All forward models and reconstruction algorithms were implemented in MATLAB. See Sec. 2 of **SI** for details.

### Tissue imaging

The sample of biological tissue that contains both fat and muscle was cut into a 1-inch by 1-inch square from the back of the pork leg, and air-dried before the experiment to prevent the sample from deformation during the imaging process. A 6mm-diameter circular ROI on the upper right corner containing both fat and muscular tissue was selected as the interior region.

### Monte Carlo simulation for radiation dose estimation

The Monte Carlo (MC) simulation on the absorbed radiation dose was performed using the commercial software *ImpactMC*^35^. The CT image of the ham sample was segmented into air, fat and muscle regions, according to the attenuation map. The mass attenuation coefficients of fat and muscle are 6.3 cm^2^/g and 10.4 cm^2^/g at 8 keV, respectively, according to the ICRU-44 standard tissue database^36^. The densities of fat and muscle are 0.95 g/cm^3^ and 1.05 g/cm^3^, respectively. The spectrum of the X-ray source (SI Fig. S9) for the MC simulations was measured with a photon counting detector (X-123, AMPTEK). The irradiance of the source was calculated using XSPECT under experimental conditions (35 kV, 40 mA).

### Data availability

The datasets generated during the current study are available from the corresponding author under reasonable request.

## Electronic supplementary material

Supplementary Information
