# Supplementary material for: X-ray diffraction tomography with limited projection information
Source: Sci Rep. 2018 Jan 11;8:522. doi: 10.1038/s41598-017-19089-w (PMC5764978; doi:10.1038/s41598-017-19089-w)
Supplement: Supplementary file 1 — Supplementary Information [file 41598_2017_19089_MOESM1_ESM.pdf]

Supplementary Information

X-ray diffraction tomography with limited projection information

Zheyuan Zhu[1], Alexander Katsevich[2], Anuj J. Kapadia[3], Joel A. Greenberg[4], Shuo Pang[1*]

[1]CREOL College of Optics and Photonics, University of Central Florida, Orlando 32816, USA

[2]Department of Mathematics, University of Central Florida, Orlando 32816, USA

[3]Department of Radiology, Duke University, Durham 27705, USA

[4]Department of Electrical and Computer Engineering, Duke University, Durham 27708, USA

*pang@creol.ucf.edu

1. Analytical description of X-ray diffraction tomography (XDT)

In the following subsections, we will establish the analytical model of X-ray diffraction tomography (XDT). In Section 1.1, we briefly describe the concept of coherent scattering, and then establish the forward imaging model between the form factor and the diffraction profile measurement (Sec 1.2). In Section 1.3, we show that under the linear approximation of the Bragg's law, the imaging model of the 2-dimensional XDT is equivalent to a 3-dimensional parallel fan beam CT problem. Finally, the truncated XDT geometry will be described in Section 1.4.

1.1 X-ray diffraction. In this section we will briefly describe the concept of coherent scattering. Besides photoelectric absorption, X-ray photons may interact with matter by two other major mechanisms: Compton incoherent scattering and Rayleigh coherent scattering. In the coherent scattering process, no energy from the X-ray is converted into the kinetic energy of the electron, yet the direction of incident photon is altered. For X-

ray diffraction, coherently scattered photons interfere with each other and form diffraction pattern. The number of diffraction photons, dI, from a small object voxel dV, in the direction of scattering angle θ_{sc} covering a small solid angle $d\Omega$ can expressed as:

$$dI = \frac{r_e^2}{2}(1+\cos^2\theta_{sc})f(\mathbf{r},q)d\Omega dVdq\ , \tag{1}$$

where $r_e = 2.82 \times 10^{-15}\ m$, is the classical electron radius. The object is described by $f(\mathbf{r},q) = n(\mathbf{r})F^2(\mathbf{r},q)$, which is the product of the density of scatter material $n(\mathbf{r})$ and the molecular form factor $F^2(\mathbf{r},q)$ at location $\mathbf{r}$. The molecular form factor $F^2(\mathbf{r},q)$ can be measured through the diffraction profile, where q is the momentum transfer. It is related to the scattering angle via Bragg's Law,

$$q = \frac{E}{hc}\sin\left(\frac{\theta_{sc}}{2}\right), \tag{2}$$

where E is the energy of X-ray photon. The constants $h = 6.63 \times 10^{-34}\ Js$ and $c = 3 \times 10^8 m/s$ are the Planck's constant and the speed of light, respectively.

1.2 Pencil beam XDT. In this section, we relate the tomographic measurement geometry with the molecular coherent scattering profile. To reconstruct an extended object with two spatial dimensions, $f(x,z,q)$, from a series of diffraction profiles, the rotation around the y-axis and translation perpendicular to the pencil beam direction are required, as depicted in Figure S1. We define the sample coordinates (x,z,q) and the measurement system coordinates (s,t,r). s is the beam offset, the distance from the rotation center to the pencil beam; t is the voxel depth along the pencil beam; and r is the distance between a detector pixel and the pencil beam.

Similar to conventional CT, the coordinate transformation at rotation angle φ between the sample coordinates and system coordinates is

$$\Gamma = \{(x_\varphi, z_\varphi) : x_\varphi = s\cos\varphi + t\sin\varphi; z_\varphi = -s\sin\varphi + t\cos\varphi\} . \quad (3)$$

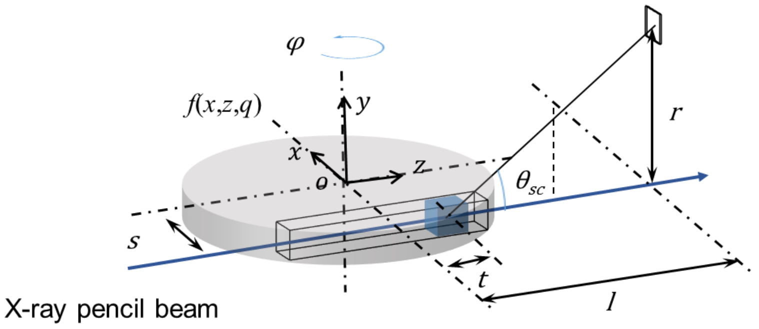

Figure S1. X-ray diffraction tomography geometry. Object $f(x,z,q)$ is translated perpendicular to the pencil beam direction s, and rotated by angle φ around the y-axis. t is the voxel depth along the pencil beam, and r is the distance between a detector pixel and the pencil beam. l is the distance between the rotation center and the detector plane. The solid, black cuboid illustrates the scattering volume, and the dark blue cube represents each voxel in our forward model.

Angular dispersive setup employs a narrow band X-ray source with a central energy at E. The rotation center to detector plane distance l is much larger than the size of the sample. At small scattering angle, $\sin\theta_{sc} \approx r/(l-t)$. Plugging it to the Bragg's Law, i.e. Equation (2), we have $q = \frac{Er}{2hc(l-t)}$. Given that the incident pencil beam cross-section area is A, the volume of each voxel $dV = Adt$. The solid angle of the detector pixel is $d\Omega = \frac{\Delta^2}{(x-r)^2 + (l-t)^2}\cos\theta_{sc}$, where Δ is the size of the detector. Under small diffraction angle approximation, $\cos\theta_{sc} \approx 1$. Equation (1) can be written as

$$I_\varphi(s,r) = Ar_e^2\Delta^2 \iint \frac{1}{(l-t)^2 + r^2} f(x_\varphi(s,t), z_\varphi(s,t), q)\,\delta\left(q - \frac{Er}{2hc(l-t)}\right) \mathrm{d}t\mathrm{d}q . \quad (4)$$

$\delta(\cdot)$ is the Dirac delta function, enforcing the Bragg's law. Equation (4) establishes the mapping from the object space (x,z,q) to the measurement space (s,r,φ). The integral

on the qt plane is evaluated along the family of curves $q - \frac{Er}{2hc(l-t)} = 0$, with the detector distance r as a parameter [1]. The XDT reconstruction problem is to recover the molecular form factor of the object $f(x, z, q)$ from a series of measurements $I_{\varphi}(s, r)$.

1.3 XDT reconstruction as parallel fan-beam CT problem. In this section we will introduce a couple of first order approximations that are instrumental in relating the 2-dimensional XDT problem with a circular cone beam 3-dimensional CT problem. Strictly speaking, the coherent scattering tomography is not a cone-beam CT, but vertical parallel fan beam projection.

In Equation (4), other than the factor $\frac{1}{(l-t)^2+r^2}$ and the Bragg's law enforced by the Dirac delta function, the transformation resembles that of 3D parallel beam projection. Since the object dimension t_{max} is on the order of $10\,mm$, which is much smaller than the detector to rotation center distance l is on the order of 10 cm. The factor $\frac{1}{(l-t)^2+r^2} \approx \frac{1}{l^2+r^2}$ can be taken out of the integral. Also since $l \gg t$, we took the first order approximation of the projection curve $q - \frac{Er}{2hc(l-t)} = 0$.

$$q = \frac{Er}{2hc(l-t)} \approx \frac{Er}{2hcl}\left(1 + \frac{t}{l}\right), \tag{5}$$

Despite being mathematically trivial, after applying both approximations, Equation (4) can be further simplified.

$$I_{\phi}(s, r) = \frac{A r_e^{2} \Delta^2}{l^2 + r^2} \int f\left[s\cos\varphi + t\sin\varphi, -s\sin\varphi + t\cos\varphi, \frac{Er}{2hcl}\left(1 + \frac{t}{l}\right)\right] \mathrm{d}t\,. \tag{6}$$

Let $\alpha_r = \tan^{-1}\left(\frac{Er}{2hcl^2}\right)$. Equation (6) shows that the line integral in the (x, z, q) plane is along the direction $(\sin\varphi\cos\alpha_r, \cos\varphi\cos\alpha_r, \sin\alpha_r)$, as shown in Figure S2. The projection ray intersects with the mid-plane ($q = 0$), at $t = -l$. The XDT reconstruction amounts to the inverse problem of a series of projections from vertical parallel fan beam. For each point that is not in the mid-plane, i.e. $q \neq 0$, the beams passing through it form a sheaf. Similar to the analogy between parallel beam and fan beam CT, we can rearrange the beams to show that the parallel fan beam geometry is equivalent to circular cone beam CT geometry. It is worth noting that circular cone-beam configuration, however, does not satisfy Tuy's condition [2], and the 3D Radon transform cannot be determined completely when projection data are acquired along a circular source–detector trajectory. Approximation algorithms have achieved satisfactory reconstruction, especially when the divergence angle is small.

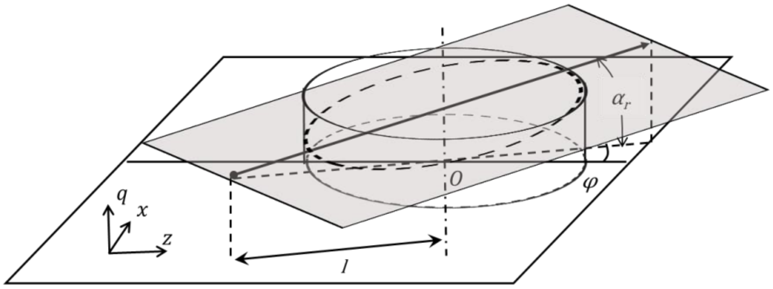

Figure S2. Illustration of the coherent scattering tomographic projection of an object $f(x, z, q)$ as a 3-dimensional parallel fan beam projection, where the projection direction deviates from the direction perpendicular to the rotation axis by an angle α_r.

In the XDT setup, the momentum transfer measurement range is between $0.030\ \text{Å}^{-1}$and $0.250\ \text{Å}^{-1}$ with step size of $0.005\ \text{Å}^{-1}$. The sampling step in the spatial domain is $0.5\ mm$. Given the object to detector distance l is $120\ mm$, the maximum angle between the mid-plane and intersection plane is $11.3°$. In Section 2, we show the reconstruction using

Feldkamp, Davis, Kress (FDK) algorithm [3]. The artefacts due to missing data in Radon space are visible in the results without regularization.

1.4 Interior XDT geometry.

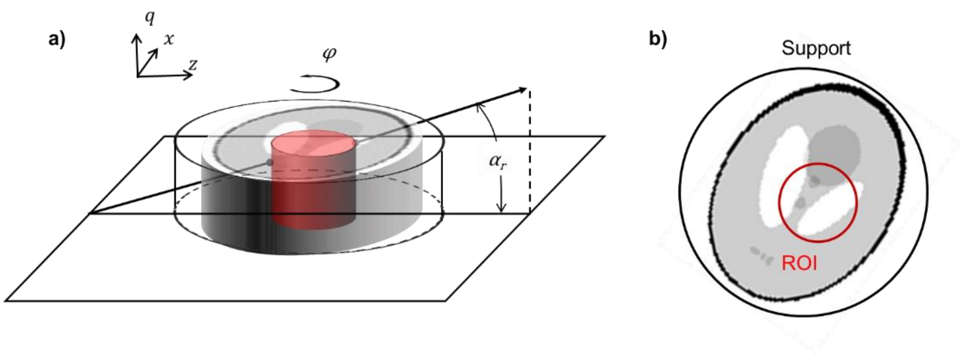

Figure S3. Interior X-ray diffraction tomographic projection. (a) Illustration of the interior X-ray diffraction tomographic projection. The red cylinder represents the area of interest. (b) Top view of the projection geometry.

For interior XDT, the object undergoes a full rotation, but the beam scans only the region of interest (ROI) that is smaller than the support of the object. From the previous section, we see that the XDT problem can be treated approximately as a cone beam reconstruction. The interior XDT problem is equivalent to a truncated cone beam problem in (x, z, q) space. The 3-dimensional geometry is shown in Figure S3.

From the perspective of filtered back projection reconstruction, the back projection is nonlocalized, and truncated cone beam problem therefore does not have unique reconstruction. Constraints and conditions that assure a unique solution of the ROI were developed from both practical and theoretical approaches. For truncation problem, much progress has been made based on the concept of differential back projection with prior knowledge of an interior region [4]. For XDT setup, to know the exact scattering profile within a region of the entire sample is usually not feasible. Based on compressed sensing theory, it has been proved that if an object under reconstruction is essentially

piecewise constant, a local ROI can be exactly and stably reconstructed based on TV regularization [5]. Then based on the forward model matrix $\mathbf{H}$, the reconstruction of object $\mathbf{f}$ is an optimization problem with TV regularization [6] [7],

$$\hat{\mathbf{f}} = \arg\min_{\mathbf{f}'}(-\log P(\mathbf{g}\,|\,\mathbf{f}') + \tau TV(\mathbf{f}')) \,, \tag{7}$$

where $P(\mathbf{g}|\mathbf{f}')$ is the Poisson likelihood of observing the measurement $\mathbf{g}$ given the parameters $\mathbf{f}'$, τ is the weight for balancing the measurement error and the TV regularizer. The TV operator is defined as

$$TV(\mathbf{f}) = \sum_{i,j,k}\sqrt{(f_{i+1,j,k} - f_{i,j,k})^2 + (f_{i,j+1,k} - f_{i,j,k})^2 + \varepsilon(f_{i,j,k+1} - f_{i,j,k})^2} \,, \tag{8}$$

Where i and j represent the index along the spatial dimensions x and z, respectively, and k represents the index along the momentum transfer dimension q. The parameter ε is a weight factor, balancing the difference of unit on the spatial and momentum transfer dimensions.

2. Simulation results

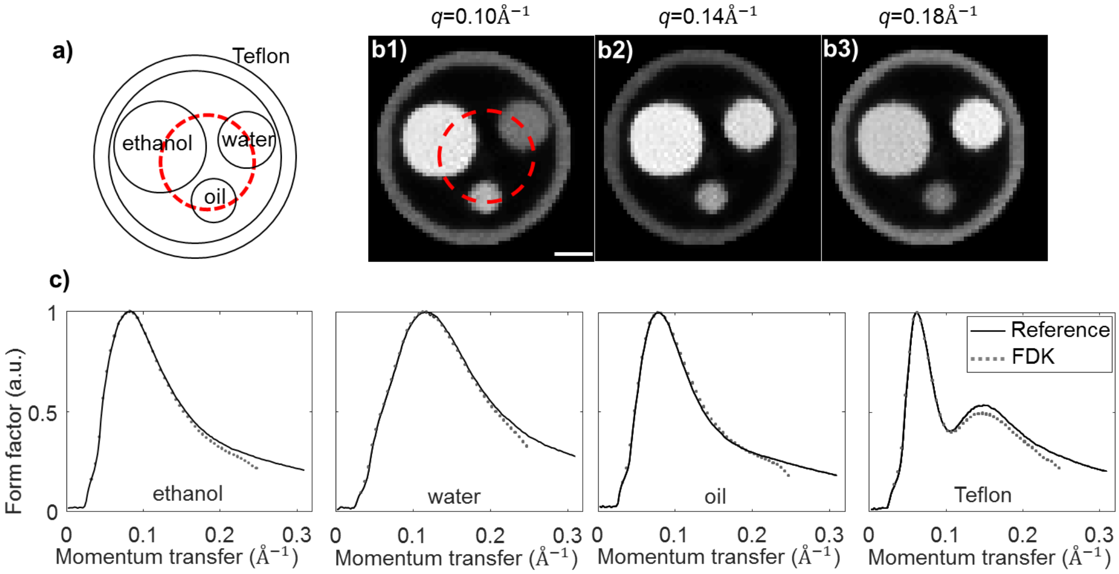

Figure S4. Simulated XDT reconstruction of the phantom. (a) The illustration of the simulation phantom. The red circle marks the interior scan region. (b) Full range XDT reconstruction at momentum transfer $0.08\,\text{Å}^{-1}$ (b1), $0.12\,\text{Å}^{-1}$ (b2), and $0.16\,\text{Å}^{-1}$ (b3). The NMSE of the reconstruction are 1.97%, 2.48% and 2.74%, respectively. (c) The form factor profiles of each material are shown and compared with the ground truth.

In this section we validate our reconstruction used in XDT reconstruction by using a simulation phantom. The reconstruction performance of full sample projection and truncated projections are evaluated. Figure S4(a) shows the simulation phantom constructed based on the geometry of the physical phantom used in the experiment. The scattering profiles of water, vegetable oil, methanol and Teflon were measured by shaping them into a $2\,mm$-wide tip. The sample was illuminated by a pencil beam, and the scattering profile was captured as the reference molecular form factors. The forward projection model has a sampling step of $0.5\,mm$ in both x and y direction, $0.005\,\text{Å}^{-1}$ in the momentum transfer space. Within the whole object, a ROI region with radius of $7\,mm$, marked by the red circle on Figure S4(a), was specified for interior reconstruction. The global XDT scan covering a full field-of-view (FOV) of 33 mm under every 2.5° rotation step. Based on the forward model, the XDT measurement of the whole sample was first calculated. The truncated projection data was extracted from the whole XDT measurement.

First, we use the FDK algorithm to reconstruct the projection of the whole sample. Figure S4(b) shows the reconstruction results at momentum transfer 0.08Å^{-1}, 0.12Å^{-1}, and 0.16Å^{-1}. The normalized mean square-root error (NMSE) of the reconstruction 1.97%, 2.48% and 2.74%, respectively. The observation of larger reconstruction error for larger q value justifies that the amount of missing information increases with larger tilt angle. The reconstructed momentum transfer profiles from four sampling point of each material are shown in Figure S4(c).

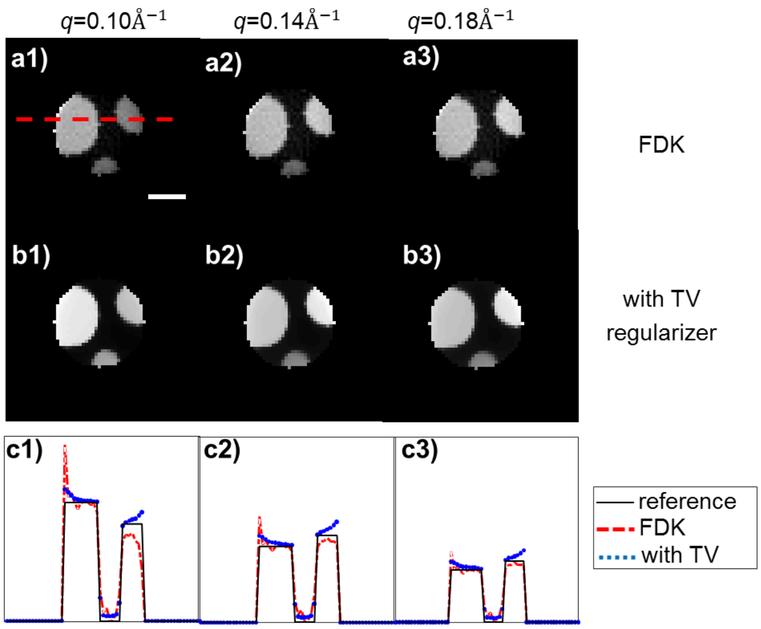

Figure S5. Simulated XDT reconstruction from truncated measurement. (a) Reconstruction using FDK algorithm at momentum transfer at momentum transfer 0.08 $\mathrm{Å}^{-1}$ (a1), 0.12 $\mathrm{Å}^{-1}$ (a2), and 0.16 $\mathrm{Å}^{-1}$ (a3). (b) Reconstruction with TV regularizer. The NMSE of the reconstruction are 1.90%, 2.51% and 2.86%, respectively. (c) Comparison of the spatial profile along the red line in (a1) between the interior reconstruction with/without regularizer and the ground truth.

Next we perform the reconstruction using FDK algorithm to the truncated projection. Figure S5(a) shows the reconstruction results at momentum transfer 0.08 $\mathrm{Å}^{-1}$, 0.12 $\mathrm{Å}^{-1}$, and 0.16 $\mathrm{Å}^{-1}$. The NMSE of the reconstruction at these momentum transfer values are 2.80%, 3.24% and 3.40%, respectively. The truncated reconstruction error is more significant than the full reconstruction due to the missing scattering information from the exterior region. The missing information has more pronounced impact near the ROI boundary. Since the simulation phantom is piecewise constant in spatial domain, from the perspective of compressed sensing, the reconstruction can be stabilized by introducing TV regularization [5]. Figure S5(b) shows the reconstruction results at momentum transfer 0.08 $\mathrm{Å}^{-1}$, 0.12 $\mathrm{Å}^{-1}$, and 0.16 $\mathrm{Å}^{-1}$. The NMSE of the reconstruction at these momentum transfer values are 1.90%, 2.51% and 2.86%, respectively, showing an average reduction of 23.7% in reconstruction error due to the regularization. The

reconstructed spatial profiles along the red, dashed line in Figure S5(a1) are shown in Figure S5(c). Comparing to the reconstruction from truncated projection using FDK algorithm, we can see that with TV regularization, the edge of the sample is preserved and the truncation errors close to the ROI boundary was suppressed.

3. Material classification

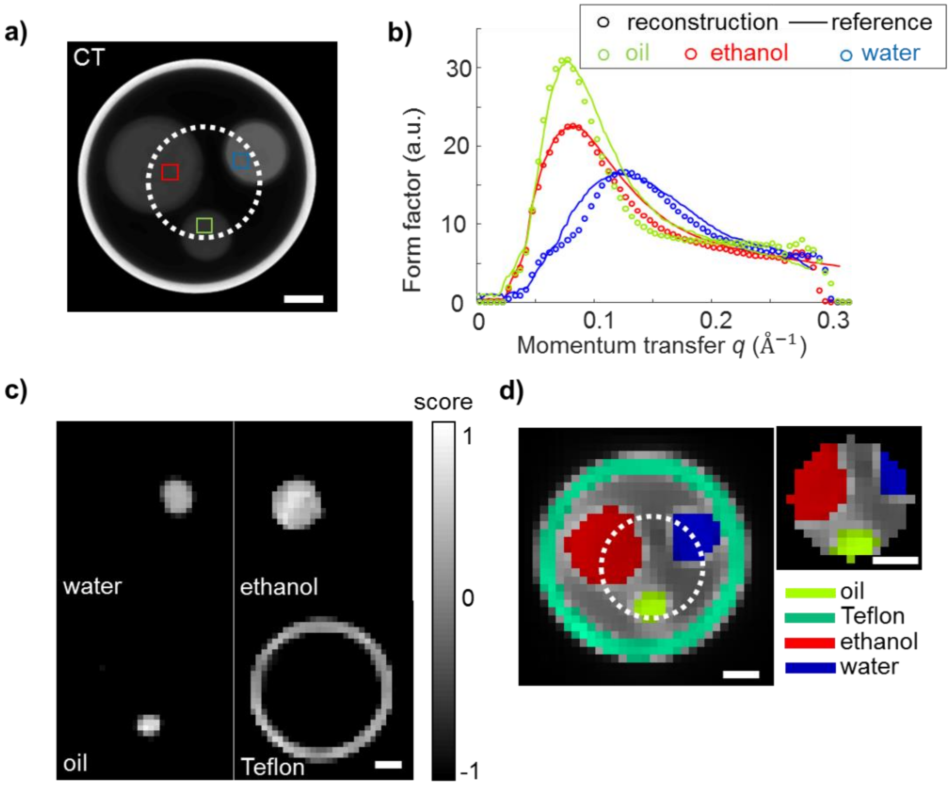

Figure S6. SVM classification of XDT and interior XDT reconstruction. (a) Attenuation map of the phantom from CT scan. (b) Reconstructed scattering profile from interior XDT measurement in the region marked by red, green and blue squares in (a), corresponding to the material of ethanol, oil, and water, respectively. (c) SVM scores of the global XDT reconstruction against the four composing materials (i.e. water, methanol, oil and Teflon). (d) Material map for the global and interior XDT. The scale bars represent 5mm.

In conventional single energy CT image, the material contrast is based on X-ray attenuation, which reflects the electron density of the material. CT images display low

contrast in liquid and soft tissues due to their similar atomic composition. XDT images reveal molecular structural information, and provide higher contrast in the momentum transfer space. In this section, we compare the material contrast between conventional CT and XDT from the perspective of material classification.

3.1 SVM classification of XDT reconstruction. The material classification on the interior XDT reconstruction was performed using support vector machines (SVM) [8]. Based on the training dataset of form factor $\mathbf{f} = \{f_{q1}, f_{q2}, ..., f_{qn}\}$, we construct a set of hyperplanes to discriminate a reconstructed form factor into a specific material class. The training set for SVM was obtained by experimental measurement. Each material was placed in a small tip and scanned with the pencil beam and 6 scattering profiles were measured as feature vectors in training. To separate the background from all four materials, the scattering profile of 6 pixels in the background on the reconstructed phantom were also included in the training set. Altogether, five binary SVMs were trained by classifying the scattering profile of each material and the background against the rest. A score $y = \mathbf{w} \cdot \mathbf{f} + \mathbf{b}$ is used in binary SVMs for evaluating the scattering profile, where $\mathbf{w}$ is the normal vector of the hyperplane and $\mathbf{b}$ is the offset. Correct classification would return a positive score if the scattering profile belongs to a specific material class, and vice versa. The SVM scores of the full-FOV XDT reconstruction against the four composing materials are shown in Figure S6(c). The material class that yields the highest posterior probability is assigned to be the material of each pixel. Figure S6(d) shows the material classification based on the experimental reconstruction of the full FOV and interior region superimposed on the total scattering intensity.

3.2 Contrast comparison between CT and XDT. To compare the material contrast between X-ray diffraction and attenuation based tomography, we convert both CT

attenuation map and XDT SVM score to a probability distribution $P(\text{class}\,|\,\mathbf{f})$ which describes the likelihood of a pixel belonging to a particular material class.

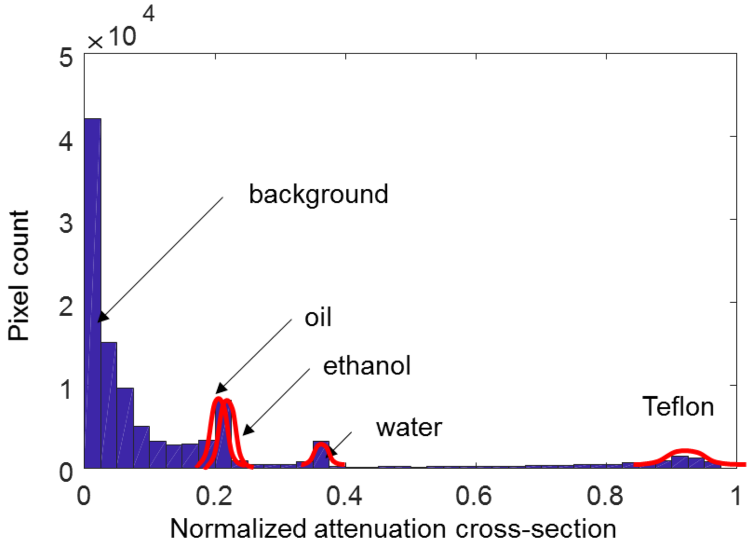

Figure S7. Image histogram of attenuation based CT image shown in Fig. S6(a). Vegetable oil and ethanol has very similar attenuation coefficients, showing as one peak in the histogram.

In CT image, the scalar attenuation value is f. The histogram of the CT image of the phantom was shown in Figure S7. The attenuation cross-section distribution of each material, $P(f|\text{class} = \text{i})$, can be modeled by a Gaussian function. We pick 20 pixels from each materials to calculate the mean μ and standard deviation σ of the grayscale value f. The results are shown in Table 1.

	Oil	ethanol	water	Teflon
**mean μ**	0.168	0.195	0.350	0.810
**std. σ**	0.034	0.034	0.028	0.156

Table1. The mean and standard deviation of the attenuation coefficient for each material.

Without any prior knowledge of the material distribution, we assume probability of each material is equal, i.e. $P(\text{class} = \text{i}) = \frac{1}{\text{number of classes}}$. The conditional probability of each pixel on the CT image belonging to a specific material class i is computed by

$$P_{CT}(\text{class} = \text{i} \mid f) = \frac{P(f \mid \text{class} = \text{i})}{\sum_j P(f \mid \text{class} = j)} \tag{9}$$

XDT images have better material contrasts in the scattering profile, rather than a single value of attenuation. To compare the material classification with attenuation based CT, the SVM score was also converted to a probability $P(\text{class} = \text{i}|\mathbf{f})$ that describes the likelihood of each scattering profile belongs to one of the four materials. Given the SVM outputs, we can fit the score y into a parametric sigmoid function,

$$P_{XDT}(\text{class} = \text{i} \mid \mathbf{f}) = \frac{1}{1 + \exp\left(A_i y_i(\mathbf{f}) + B_i\right)} \tag{10}$$

where the parameters A_i and B_i are obtained by maximizing likelihood function [9]. The probability of belonging to each class $P(\text{class} = \text{i}|\mathbf{f})$ was evaluated using the fitted parameters at each pixels. Figure S8 compares the probability of each material in the Region 1-3 defined in Figure 2.

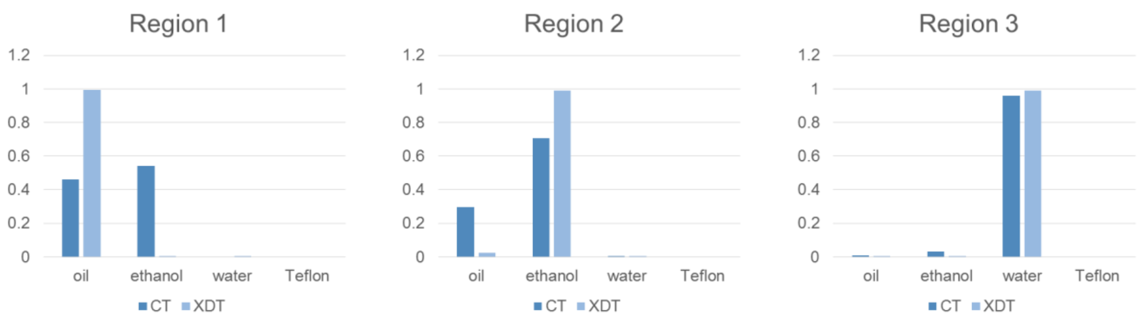

Figure S8. Probability of material in each highlighted region marked by the numbers in (b). The probability is obtained by converting the distribution of SVMs scores to a posterior probability.

4. X-ray spectrum

The spectrum of our X-ray pencil beam is measured using a photon-counting detector (X-123, AMPTEK). To estimate the source irradiance, we also simulated the spectrum of a copper-anode tube operating under 35kV using XSPECT. The measured spectrum was then scaled up so that the total number of photons matches that on the simulated spectrum. Fig. S9 shows the spectrum of our source used in the Monte Carlo simulation.

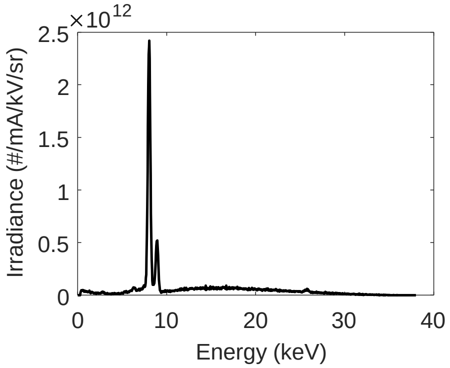

Figure S9. Spectrum of the source used in Monte Carlo simulation. The spectrum was measured experimentally and then scaled according to the total irradiance in the XSPECT simulation.

References

[1] S. Pang, Z. Zhu, G. Wang, and W. Cong, “Small angle scatter tomography with a photon counting detector array,” *Phys. Med. Biol.*, vol. 61, no. 10, pp. 3734–3748, 2016.

[2] H. K. Tuy, “An Inversion Formula for Cone-Beam Reconstruction,” *SIAM Journal on Applied Mathematics*, vol. 43. pp. 546–552, 1983.

[3] L. A. Feldkamp, L. C. Davis, and J. W. Kress, “Practical cone-beam algorithm,” *J. Opt. Soc. Am. A*, vol. 1, no. 6, p. 612, 1984.

[4] H. Kudo, M. Courdurier, F. Noo, and M. Defrise, “Tiny a priori knowledge solves the interior problem,” *IEEE Nucl. Sci. Symp. Conf. Rec.*, vol. 6, pp. 4068–4075, 2007.

[5] H. Yu and G. Wang, “Compressed sensing based interior tomography.,” *Phys. Med. Biol.*, vol. 54, no. 9, pp. 2791–2805, May 2009.

[6] W. H. Richardson, “Bayesian-Based Iterative Method of Image Restoration,” *J. Opt. Soc. Am.*, vol. 62, no. 1, p. 55, Jan. 1972.

[7] A. Chambolle, “An Algorithm for Total Variation Minimization and Applications,” *J. Math. Imaging Vis.*, vol. 20, no. 1, pp. 89–97, 2004.

[8] C. Cortes and V. Vapnik, “Support-Vector Networks,” *Mach. Learn.*, vol. 20, no. 3, pp. 273–297, 1995.

[9] J. Platt, “Probabilistic outputs for support vector machines and comparisons to regularized likelihood methods,” *Adv. large margin Classif.*, vol. 10, no. 3, pp. 61–74, 1999.
